# Multitasking roles of mosquito labrum in oviposition and blood feeding

**DOI:** 10.3389/fphys.2015.00306

**Published:** 2015-10-29

**Authors:** Young-Moo Choo, Garrison K. Buss, Kaiming Tan, Walter S. Leal

**Affiliations:** Department of Molecular and Cellular Biology, University of California-DavisDavis, CA, USA

**Keywords:** proboscis, oviposition attractant, 4-ethylphenol, CquiOR99, olfaction

## Abstract

Reception of odorants by two main head appendages, antennae and maxillary palps, is essential for insects' survival and reproduction. There is growing evidence in the literature suggesting that the proboscis is also an olfactory appendage and its function as an additional “antenna” has been previously proposed. We surmised that movements of the labrum toward a blood vessel might be chemically oriented and, if so, there should be odorant receptors expressed in the labrum. To test this hypothesis, we first compared by quantitative PCR expression of odorant receptors (OR) from the Southern house mosquito, *Culex quinquefasciatus* in antennae and proboscis and, subsequently compared OR expression in various proboscis parts. Our data suggested that a receptor for the oviposition attractant, skatole, CquiOR21, was not expressed in proboscis, whereas a receptor for another oviposition attractant, 4EP (4-ethylphenol), CquiOR99, and a receptorf for the insect repellent DEET, CquiOR136, were expressed in the stylet of the proboscis, particularly in the tip of the labrum. In a dual-choice olfactometer, mosquitoes having the stylet coated with nail polish were attracted to 4EP in the same manner as the untreated mosquitoes. By contrast, in an oviposition assay, the stylet-treated mosquitoes did not discriminate 4EP from control oviposition cups, whereas the untreated mosquitoes (as well as mosquitoes having the labella coated) laid significantly more egg rafts in cups treated with 4EP. Ablation experiments confirmed that 4EP was sensed by the labrum where CquiOR99 is highly expressed. Stylet-coated, labella-coated, and untreated mosquitoes laid significantly more egg rafts in skatole-treated cups than in control cups. Likewise, coating of proboscis structures with nail polish had no effect on DEET-mediated oviposition deterrence. In a behavioral arena designed to mimic a human arm, mosquitoes showed significantly reduced probing time when blood was impregnated with 4EP, i.e., they engaged more rapidly in continuous blood feeding as compared to untreated blood. The time of engagement for feeding in skatole-containing blood vs. untreated blood did not differ significantly. Taken together, these data suggest that 4EP reception by the labrum is important not only for oviposition decisions, but also for reducing probing and initiation of blood feeding.

## Introduction

For survival and reproduction, female mosquitoes must feed on plants to replenish energy-rich compounds they use as fuel for flight, obtain nutrients for reproduction through blood meals from vertebrate hosts, and, subsequently, lay fertilized eggs on environments suitable for their offspring to flourish. The gustatory system and, more importantly, the olfactory system are crucial for mosquito fitness in the environment. Three major head appendages are involved in reception of chemical signals and chemical cues from the environment, namely, the antennae, maxillary palps and proboscis. Although it has been well-established that antennae and maxillary palps are the main olfactory organs, recently it has been suggested that the proboscis might possess functional similarity with other typical sensory organs, i.e., the proboscis may function as an “antenna” in addition to the canonical antennae and maxillary palps (Maekawa et al., 2011). Indeed, Kwon and collaborators (Kwon et al., 2006) demonstrated that as many as 24 odorant receptor (*OR*) genes are expressed in the proboscis of the malaria mosquito, *Anopheles gambiae*, thus inferring that the proboscis might have an olfactory function. Additionally, it has been demonstrated that ablation of the entire proboscis drastically reduces the mosquitoes' ability to find a host from a short distance (Maekawa et al., 2011).

**Figure 1:**
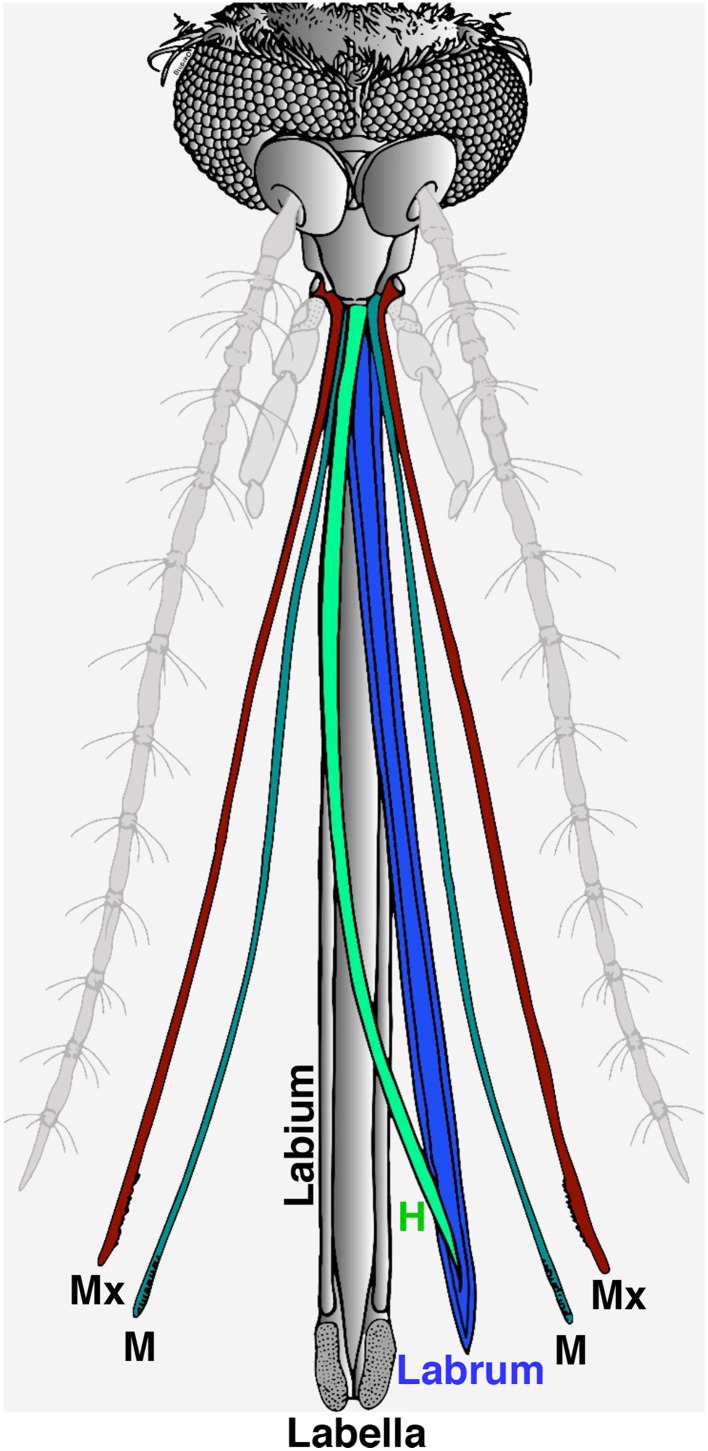
**Illustration of head appendages from the Southern house mosquito**. In this computer reconstruction, the two main olfactory appendages, the antennae and the maxillary palps, were de-emphasized in light gray. For clarity, the proboscis is depicted with the stylet removed from the labium, and the six fascicles forming the stylet separated and displayed in color. During blood feeding, the labium (in gray) bends and remains outside with the two lobes on the tip (labella) making direct contact with the host's skin. All six fascicles of the stylet are inserted in the host's skin, i.e., a pair of maxillae (Mx, cayenne), a pair of mandibles (M, fern), the hypopharynx (H, clover), and the labrum (blueberry).

The proboscis is a sophisticated “microneedle system” (Kong and Wu, 2010), which is comprised of a gutter-like labium that encloses a fascicle. There are two lobes (labella) at the tip of the labium and the fascicle contains six stylets: a pair of teeth-bearing maxillae, a pair of mandibles, a hypopharynx with its salivary canal, and a labrum that carries sense organs on its tip (Figure 1; Wahid et al., 2003). During feeding, the fascicle penetrates the host's skin while the labium bends and the labella remain in direct contact with the outer layer of the skin. It is believed that the “nanosharp tips” of the labrum and the microsawteeth of the maxillae exhibit a unique cooperative motion that enables the mosquito fascicle to painlessly penetrate the skin, with three orders of magnitude smaller force than the minimal force required for artificial microneedles to penetrate into the human skin (Kong and Wu, 2010). It is well-known that the labella house olfactory and gustatory sensilla (Sparks et al., 2013, 2014; Sparks and Dickens, 2014), but out of the six stylets, the labrum is the only one known to carry sense organs (Lee, 1974).

It has been suggested earlier on that if a female mosquito standing on human skin were to randomly pierce and try to suck, “she would have a low probability of finding blood because blood vessels represent only 1–2% of the skin volume” (Werner-Reiss et al., 1999). A recent elegant videomicroscopy analysis of mosquito blood feeding on mouse skin showed that penetration may happen immediately after the labella is brought into contact with the skin, but in some cases the proboscis may be moved over the mouse skin for some time before the fascicle penetrates the skin (Choumet et al., 2012). More interestingly, it was clearly shown that upon skin penetration, the labrum bends and makes apparently oriented movements toward blood vessels (Choumet et al., 2012) thus suggesting that reception of volatile chemicals might be important for labrum orientation. The engorgement step begins immediately after insertion of the labrum into a blood vessel (capillary feeding) or by drawing blood from a hemorrhage (pool feeding). It is not yet known if there are short-range chemicals mediating orientation of the labrum inside the skin, although it has been demonstrated that labral apical sensilla respond to phagostimulants (Liscia et al., 1999; Werner-Reiss et al., 1999), but this would require direct contact with the substrate. We surmised that movements of labrum toward blood vessels could be chemically mediated by volatile compounds. Of note, human blood is rich in volatile aromatic compounds, including phenylacetic acid, 4-ethylphenol and benzoic acid (Loke et al., 2009). To test this hypothesis, we employed a reverse chemical ecology approach to identify if odorant receptors from the Southern house mosquito, *Culex quinquefasciatus*, are expressed in the labrum and inferred their role by behavioral observations.

## Materials and methods

### Insects

*C. quinquefasciatus* mosquitoes used in this study originated from a stock laboratory colony, which in turn started from adult mosquitoes collected in Merced, CA in the 1950s and is maintained by Dr. Anthon Cornel in the Kearney Agricultural Center, University of California. In Davis, mosquitoes were maintained in an insectary at 27 ± 1°C, under a photoperiod of 12:12 h (L:D) for the last 5 years. Two blood meals were provided to females on the day 3–4. Four days after the first blood meal, the blood-fed females were used for oviposition bioassays, whereas non blood-fed mosquitoes were used for a modified surface landing and feeding assays (Xu et al., 2014).

### Quantitative analysis of transcription levels

For qRT-PCR, each type of tissue (antennae, proboscis, stylet, labium, hypopharynx-maxilla-mandibles, labrum tip, labrum without tip, labella, and labium without labella) from a minimal of 500 blood-fed female mosquitoes (4–5 days post blood meal; 8–9 days old) was dissected and collected in TRIzol reagent (Invitrogen, Carlsbad, CA) on ice using a stereo microscope (Zeiss, Stemi DR 1663, Germany). Total RNA was extracted using TRIzol reagent. After RNA was quantified on NanoDrop Lite spectrometer (Thermo scientific, Rockford, IL), cDNA was synthesized from total RNA (500 ng of equal amount RNA from antennae vs. proboscis; 100 ng of equal amounts of RNA from other small tissues; stylet vs.labium, labrum vs.hypopharynx-maxilla-mandibles mixtures, and labella vs.labium without labella; 25 ng of equal amounts of RNA from labrum tip vs.labrum without tip) using iScript™ Reverse Transcription Supermix for RT-qPCR according to the manufacturer's instructions (Bio-Rad, Hercules, CA). Real-time quantitative PCR (qPCR) was carried out by using a CFX96 TouchTM Real-Time PCR Detection System (Bio-Rad) and SsoAdvanced SYBR Green Supermix (Bio-Rad). *CquiRPS7* gene was used as reference. We designed a pair of detection primers for each of the following genes by Primer 3 program (http://frodo.wi.mit.edu/):

CquiOR1-Fw: 5′-TCCGGAAAGGAAGATCATTG-3′;
Rv: 5′-CGTTACAAACTCGGGACGAT-3′;
CquiOR21 (= CquiOR10 Hughes et al., 2010)-Fw: 5′- TGCATCGAAGACCACAAGAG -3′;
Rv: 5′- TCATGGAATCGTCCAGTTCA -3′;
CquiOR37-Fw: 5′-GGTTCTTATGGGCGAGATGA-3′;
Rv: 5′-TACGAGTACGACGCTTGCAC-3′;
CquiOR44 -Fw: 5′-AGTGGCACAGTGAGATGCAG-3′;
Rv: 5′-CACCTCGAGCAGAAACATCA-3′;
CquiOR55-Fw: 5′-CACGTGGAATTGGTTCTCCT-3′;
Rv: 5′-TAGATTTTGGCCAACCCTTG-3′;
CquiOR64-Fw: 5′-GTTCCAGGAGGAAACAGCAG-3′;
Rv: 5′-CCAAGAAAACCGGTCGATAA-3′;
CquiOR73-Fw: 5′-CTGGGTATGCTGAGGAACTTC-3′;
Rv: 5′-GCAGCCAGATCCAAAAGTTG-3′;
CquiOR93-Fw: 5′-AGCTTGATCGCTGAATCGTT-3′;
Rv: 5′-ATGATCATGGCGAGGAACTC-3′;
CquiOR95-Fw: 5′-AAGTCATCCGGCAATTTTTG-3′;
Rv: 5′-CAGCGATTGGTCGTTATCCT-3′;
CquiOr99-Fw: 5′- GTCATCCAGTACGGGGCTAA -3′;
Rv: 5′- AGCAAACGGTCTCTTCCAGA -3′;
CquiOR121 (=OR2)-Fw: 5′-GAGCGGCTTCACCAGTTTAG-3′;
Rv: 5′-TCCCAAACGATAGCAACTCC-3′;
CquiOR125-Fw: 5′- GGTGGACACAAGGAGGAGAA-3′;
Rv: 5′-GCGACGTATCCGTCCAAATG-3′;
CquiOR132-Fw: 5′-ACGATCCTTTGCAGCAACTT-3′;
Rv: 5′-TCAACGGTGAGCACTTTGAG-3′;
CquiOR136-Fw: 5′-CAACGCTCGCAAATATCTCA-3′;
Rv: 5′-TGAGCACTCGCCATTTGTAG-3′;
CquiOR151-Fw: 5′-TGAGCTCATATTGCGGAGTG-3′;
Rv: 5′-TTGGCCAGAATTTCCTGTTC-3′;
CquiOR161-Fw: 5′-GTCCAGAGCTGGATCCTCAG-3′;
Rv: 5′-AGCGAAAAGGCAAAGTTGAA-3′;
CquiRPS7-Fw; 5′-ATCCTGGAGCTGGAGATGA -3′;
Rv: 5′-GATGACGATGGCCTTCTTGT -3′.

Reactions were run with the following standard program: 95°C for 30 s, 39 cycles of 95°C for 5 s, 55°C for 10 s, 72°C for 30 s, melt curve of 65 to 95°C, increment 0.5°C, 5 s. The data were analyzed using the 2^−Δ*ΔCT*^ method by Bio-Rad CFX Manager 2.1 software. qPCR was performed with 3 biological replicates, each having 3 technical replicates.

### Behavioral assays

Oviposition bioassays were performed as previously described (Zhu et al., 2013) using collapsible Field Cages (Bioquip: 30.5 × 30.5 × 30.5 cm). Cage covers were extensively cleaned up with hot water before and after tests. In each cage two falcon specimen container (Product #354014; Corning, NY, USA) filled with 100 ml of distilled water were placed in opposite corners. Final concentrations of 4-ethylphenol (1 μg/L), skatole (10^−3^μg/L), and DEET (0.01%) were diluted in 100 ml water, whereas the same volume of ethanol was added for control water. Two to four cages were used for oviposition behavior assay per day. Thirty gravid females per cage (4 days after the first of two blood meals) were used for oviposition assays during scotophase. Egg rafts in treated and control water were counted the following morning, cages were changed, and specimen containers were rotated for the next replicate with a fresh group of gravid females. The data were analyzed by two-tailed paired sample *t*-test (GraphPad Prism 6, La Jolla, CA).

**Figure 2:**
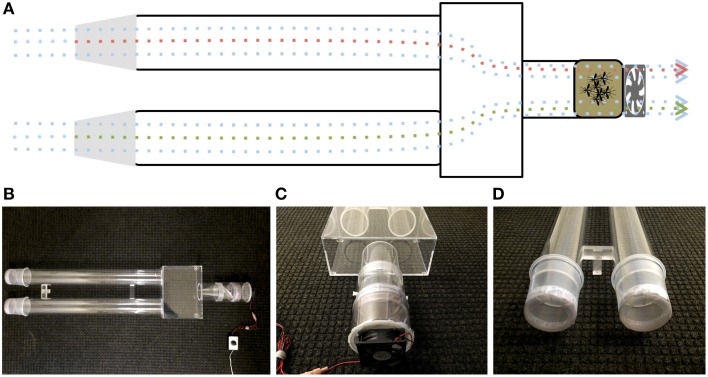
**Diagram and photographs of the dual-choice olfactometer. (A)** Diagrammatic representation of the olfactometer highlighting the suction system and the direction of the plume. **(B)** Bird-view of the olfactomer and photos illustrating **(C)** the suction system and release chamber and **(D)** the sample compartments at the upwind end of olfactometer.

Upwind attraction was measured using a customized dual-choice olfactometer (Figure 2), prepared in Plexiglas® with small modifications of previously described olfactometers (Geier et al., 1999; Bosch et al., 2000; Cooperband et al., 2008). A Plexiglas® box (13 cm high, 30.1 cm wide, and 21 cm long) was permanently connected to two Plexiglas® cylinders (79.7 cm long, 9.2 cm internal diameter) and one shorter Plexiglas® cylinder (11.2 cm long, 9.2 internal diameter) at the downwind end. Mosquito release chambers (11 cm long, 10.2 internal diameter) had a wire mesh, rotating door in the middle of the chamber (Geier et al., 1999), a polished end to connect to the downwind end of the olfactomer and the other end covered with Bioquip's field mosquito cage cover (1451BC) to connect to a small plastic cylinder (7.8 cm long, 11.2 cm internal diameter), which housed a computer fan (Comair Rotron, Flight® LT, 12 VDC, 0.45A). The downwind ends of the olfactometer were capped with ice cream cups (Solo Foodservice, Lake Forest, IL: 10.8 cm long, 10.5 cm internal diameter). The bottom of these cups (8.9 cm internal diameter) were removed and replaced with the above-described mosquito cage cover. Filter paper pieces (2 × 2 cm), either treated with 4-ethylphenol (4EP, Sigma-Aldrich) or hexane only, were hung at the center of the cups by wire holders, which were placed 2 cm away from the end of the cups. At dusk, a release chamber was combined with release arm of the olfactometer and mosquitoes were allowed to acclimate for 10 min in the dark room at 27 ± 2°C. The release chamber was connected to the compartment housing the fan, which generated an airflow of 7 ± 1 cm/s measured at upwind end of each arm of the olfactometer. While the mosquitoes adjusted to the dark room, 4EP (10 μl of 0.1 μg/L to the middle of a filter paper strip) and 10 μl of hexane (control) on another, the ice cream cups were connected to the ends of the olfactometer. Then, mosquitoes were released and after 5 min the number of mosquitoes in treatment and control ends of the olfactometer were counted and recorded. Soon after wind tunnel experiments, mosquitoes were gently retrieved using a manual aspirator, and the recovered mosquitoes were used for subsequent oviposition bioassays.

The previously described surface-landing assay (Xu et al., 2014) was modified to measure the effect of 4EP on blood feeding behavior. The arena was converted from a two-port, two-choice assay into a single-port, two-choice assay by placing both dental cotton rolls 180 degrees apart (top and bottom) on a single Dudley tube, each roll held in place by a syringe needle (16 Gauge, Sigma-Aldrich) placed 1 cm away from the perimeter of the tube. For physical stimuli, water at 37°C was circulated inside of the Dudley tube, which was painted black on the internal surface. Chemical stimuli were provided by stream of CO_2_ at 50 ml/min and dental cotton rolls impregnated with defibrinated sheep blood (University of California-Davis, Biological Media Services, Cat#4024), which were placed on the top and bottom of a Dudley tube. For each test, the cotton roll was loaded with either 100 μl blood for control or 100 μl blood plus 1 μl of solutions of 4EP or skatole at various doses. Mosquito activity was recorded for 5 min with a camcorder equipped with Super NightShot Plus infrared system (Sony Digital Hanycam, DCR-DVD 810). Reviewing the tape, females were attracted to blood and started perching. Then after a short while they began inserting their proboscis into the cotton roll in order to taste the blood. This is the period of time called “start of probing” (Choumet et al., 2012). Probing itself takes a few seconds and some individuals finally remain motionless, thus indicating the beginning of the engorgement phase. Here, we defined the period of time between the start of probing and the start of feeding as “time of engagement.” This time of engagement is, therefore, different from the start of probing and probing duration, which can be measured starting from the searching period prior to probing.

### Coating sensory tissues and microsurgery

Four days post blood meal gravid mosquitoes were collected in a plastic cup and kept on ice for 30 min until they were fully immobilized. The gravid mosquito's stylet was separated from proboscis using BioQuip insect pin (size # 000; BioQuip Products, CA, USA) on glass Petri dishes filled with ice under a microscope. Attempts to coat proboscis parts with dental wax were unsuccessful. It solidified too quickly and, even when successfully applied, mosquitoes were able to remove it by grooming. Success rate was very high with nail polish (Maeno et al., 2011; van Bergen et al., 2013). The nail polisher (Sally Hansen, Advanced Hard as Nails, #2103 Clear) was applied through a syringe with a sharpened needle to the tip of a stylet or labella (tip of the labium). Thirty of these treated gravid mosquitoes were kept inside a release chamber for wind tunnel assay, which was placed in an insectary at 27°C and 80% relative humidity for at least 5 h before behavioral measurements started. They were first used in the olfactometer and subsequently tested in oviposition assays. For labrum ablation, the labrum was separated from the stylets (Figure 1), and then labrum tip was dissected using microsurgical scissors (World Precision Instruments, Sarasota, FL). Similar microsurgery was performed to remove the tip of the labium (labella; Figure 1).

## Results and discussion

Given the growing evidence in the literature suggesting that the proboscis might function as an “alternative antenna,” (Maekawa et al., 2011), we first aimed at investigating whether previously identified ORs from the Southern house mosquito were expressed in proboscis. Thus, we carried out quantitative PCR analysis to compare transcript levels of *OR* genes in antennae and proboscis. We focused on the DEET receptor, CquiOR136 (Xu et al., 2014), receptors for oviposition attractants, i.e., skatole, CquiOR21 [formerly named CquiOR10, (Hughes et al., 2010)], 4EP, CquiOR99 (Zhu et al., 2013), 4-methylphenol, CquiOR37 (Zhu et al., 2013), and indole, CquiOR121 [formerly CquiOR2, (Pelletier et al., 2010)], generic receptors, CquiOR1 (Xu et al., 2013), a terpenoid receptor, CquiOR44 (Xu et al., 2013), an eugenol receptor, CquiOR73 (Xu et al., 2013), a receptor for a natural repellent, CquiOR95 (Leal et al., 2013), a silent receptor, CquiOR161 (Xu et al., 2013), and six ORs, which are among the most expressed *OR* genes in antennae, but when their proteins were co-expressed along with CquiOrco in *Xenopus* oocytes no response was observed even when challenged with a large panel of ligands (Xu et al., 2014). Specifically, transcripts of the following “silent receptors” were quantified: CquiOR55, 64, 93, 125, 132, and 151.

**Figure 3:**
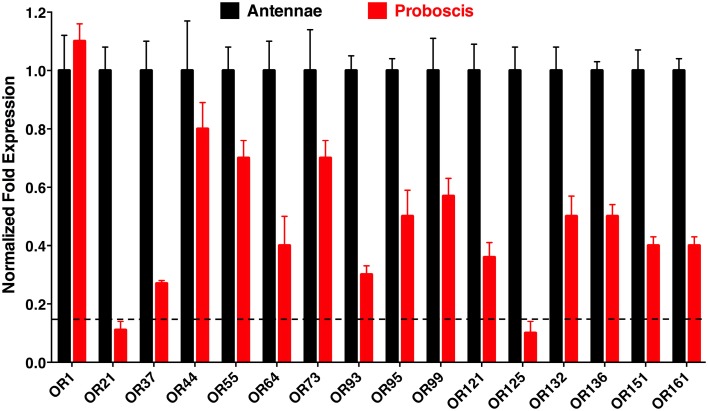
**Differential expression of odorant receptors in antennae and proboscis**. These qPCR data showed that, as expected, most receptors are more expressed in antennae than in proboscis. However, transcripts of multiple *ORs* were detected at high levels in proboscis, whereas two *OR* genes, *CquiOR21* and *CquiOR125*, appeared below an arbitrary threshold of 15%, which is depicted by a horizontal dashed line.

Quantitative PCR (qPCR) analysis demonstrated that transcript levels of the tested ORs are higher in antennae than in proboscis, except for the case of the generic receptor, CquiOR1, whose transcript levels were nearly equivalent in antennae and proboscis (Figure 3). Transcript levels for two *OR, CquiOR21*, and *CquiOR125*, were below a threshold of 15%, arbitrarily considered as “basal expression” due to unavoidable tissue cross contamination during RNA extraction. Thus, it is safe to assume that there is no significant expression of the skatole and a silent receptor, CquiOR21 and CquiOR125, respectively, in the proboscis. By contrast, transcript levels for a receptor for another oviposition attractant, 4EP, *CquiOR99*, was detected at high levels in proboscis (Figure 3). It is worth mentioning that ORs for other oviposition attractants, nonanal and trimethylamine, remain elusive. However, on the basis of high transcript levels of the 4EP receptor and basal levels of the skatole receptor, one may argue that reception of oviposition attractants by the proboscis is not a common feature of mosquito biology. We noticed, however, that upon landing on water, gravid mosquitoes make proboscis contact with the substrate, but our observations cannot dissect whether mosquitoes were drinking water (Weber and Tipping, 1993) and/or tasting the substrate. Of the other highly expressed ORs in proboscis, only two respond to compounds, which elicit clear behavioral responses. Specifically, CquiOR136 has been demonstrated to be a DEET receptor and CquiOR95 was sensitive to two repellents, ethyl 2-phenylacetate and citronellal (Leal et al., 2013). The other receptors with significant transcript levels in proboscis, particularly, CquiOR1, CquiOR44, CquiOR55, CquiOR64, CquiOR73, and CquiOR132, are either sensitive to compounds that *per se* do not elicit a clear behavioral response, or insensitive to all compounds tested to date.

**Figure 4:**
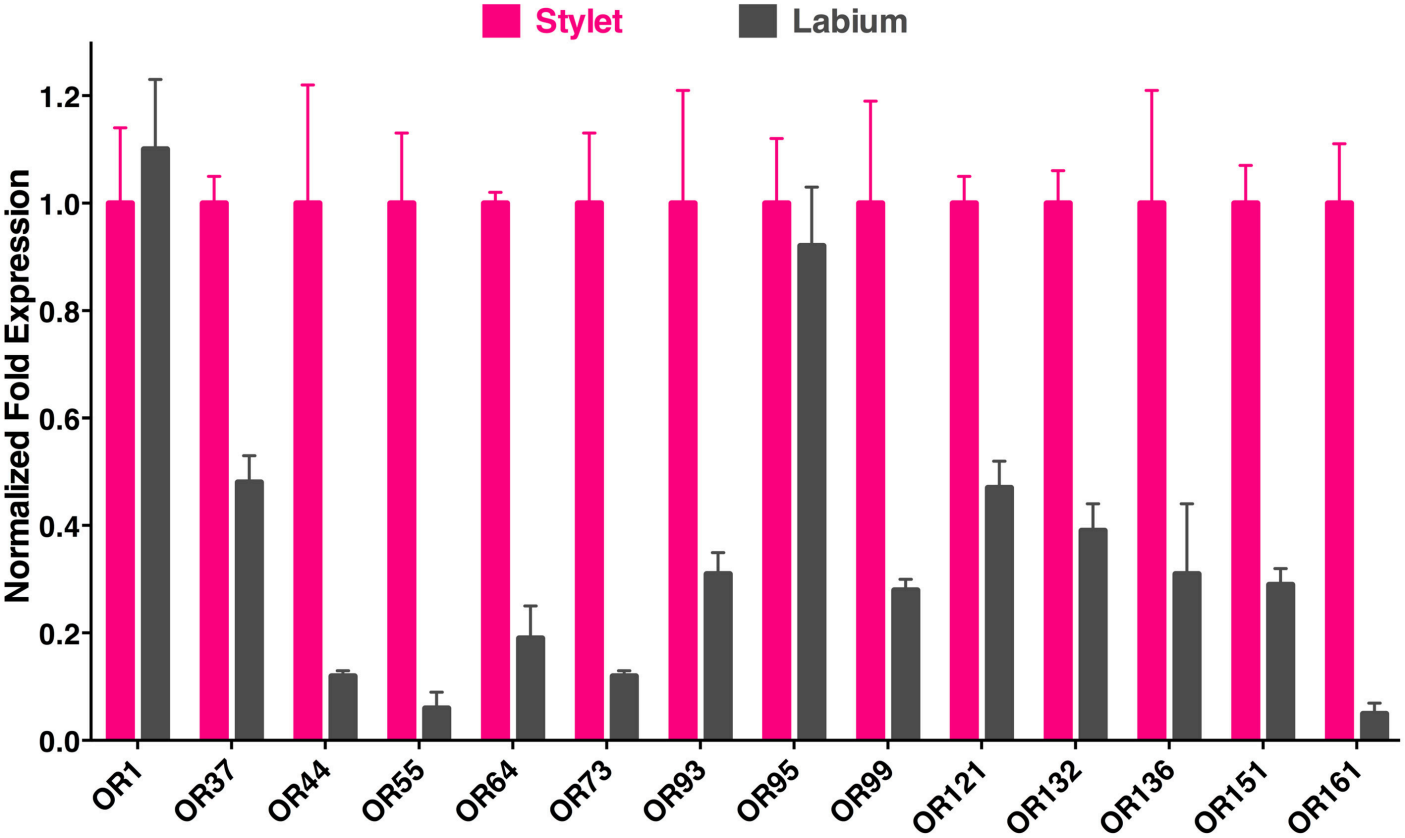
**qPCR data comparing expression of ORs in stylet and labium**. Transcript levels for most of the receptors tested were much higher in the stylet than in labium, expect for *CquiOR1* and *CquiOR95*. Of note, a DEET receptor, CquiOR136, and a receptor for the oviposition attractant, 4EP, CquiOR99, were predominantly expressed in the stylet.

**Figure 5:**
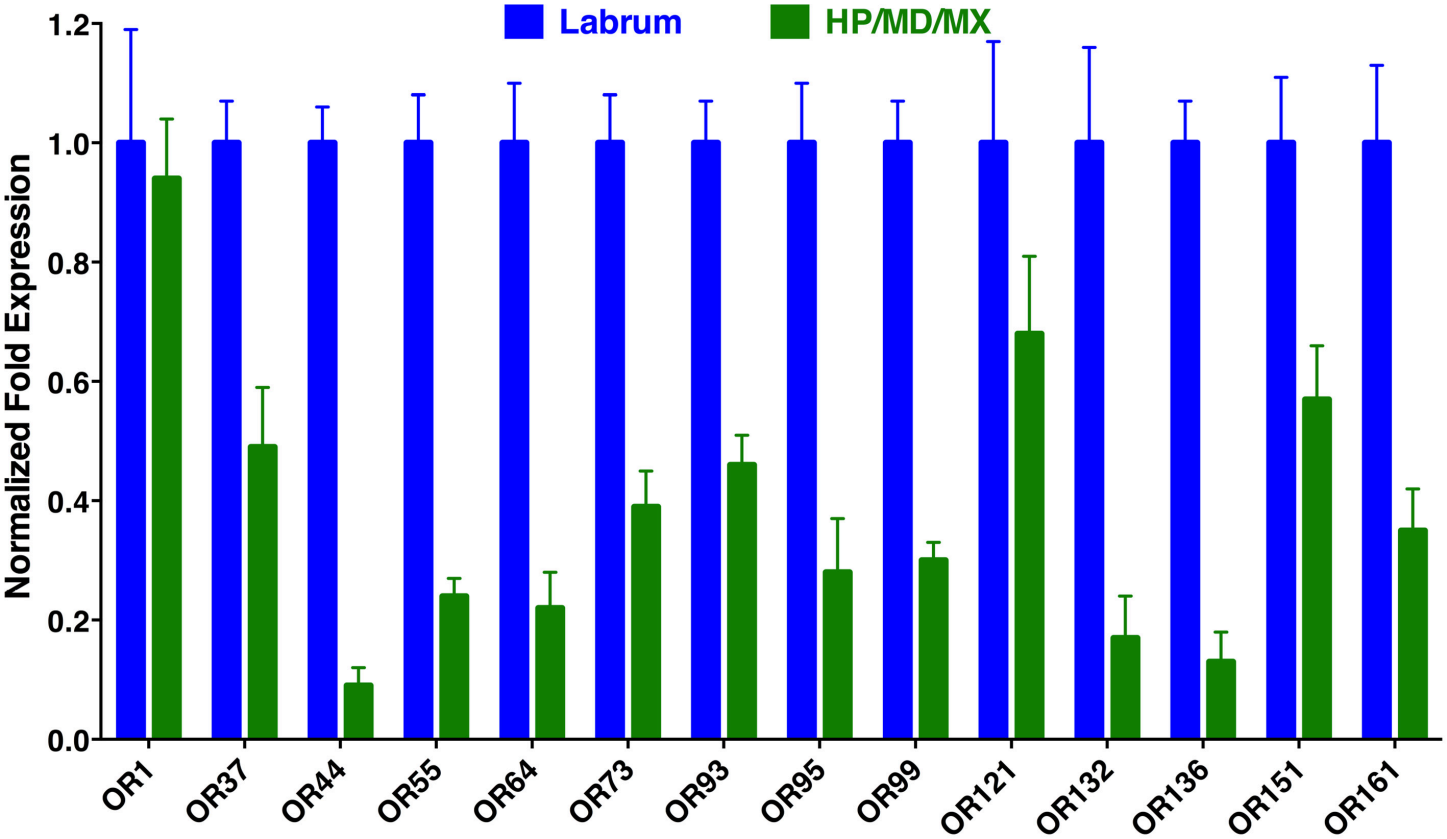
**Differential expression of ***Culex ORs*** in labrum vs. the remainder of the stylet**. As expected, transcript levels for most ORs were mainly detected in the labrum, with a notable exception of *CquiOR1*.

**Figure 6:**
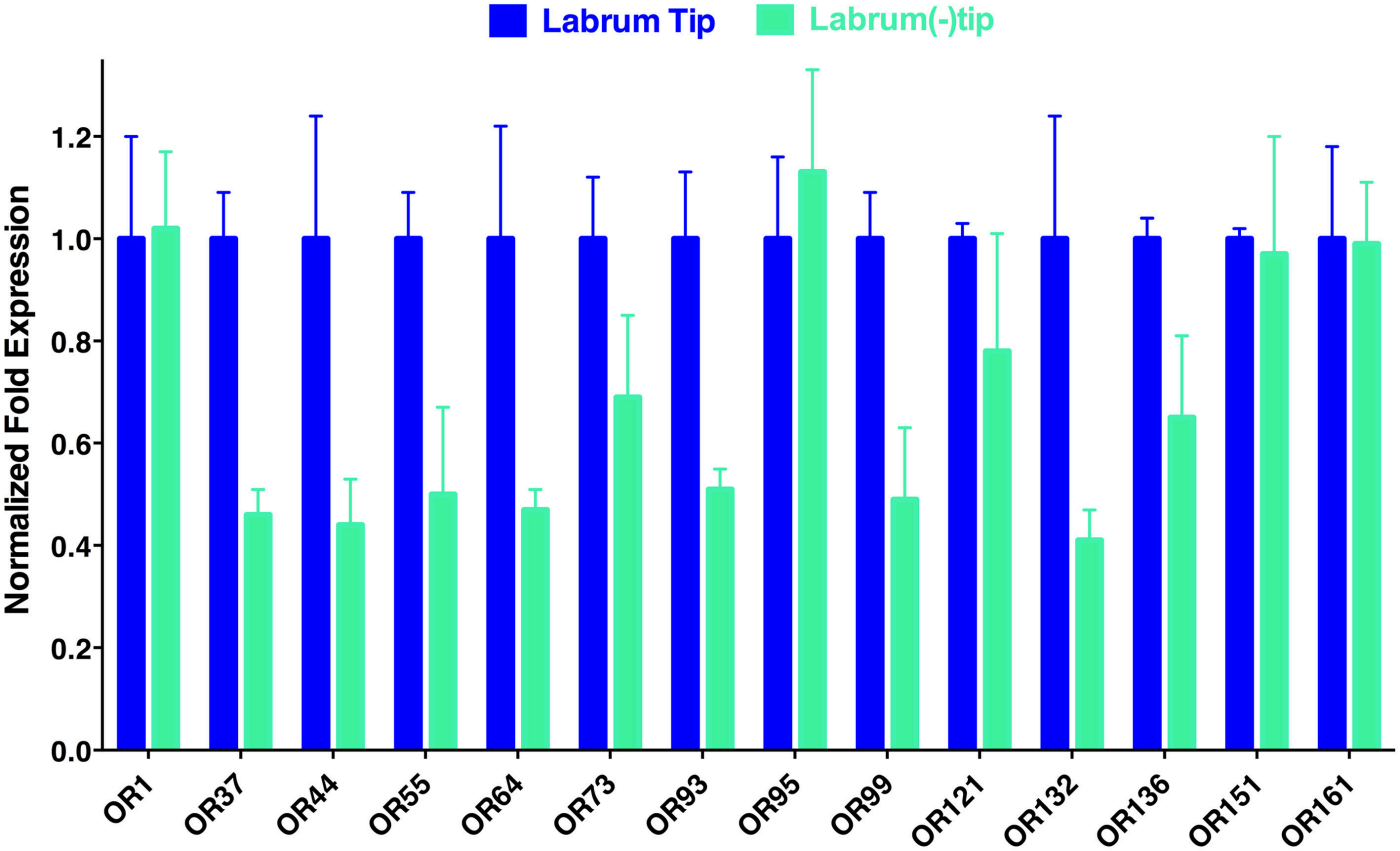
**Distribution of ORs within the labrum**. In agreement with the existence of apical and subapical sensory organs in the labrum, qPCR data suggest that the tested receptors are expressed not only on the tip, but also in the remainder of the labrum.

To determine the location of these receptors within the proboscis, we separated the fascicles (six stylets) from the labium (Figure 1), prepared qPCR samples, and compared *OR* expression in these two structures. Considering the growing evidence for expression of OR in labella (tip of the labium; Sparks et al., 2014), we were surprised to observe in general higher transcription levels in stylets rather than in labium (Figure 4), notable exceptions being transcript levels for *CquiOR1* and *CquiOR95*, whose proteins have been demonstrated to be a “generic” receptor (Xu et al., 2013) and a receptor sensitive to a natural repellent (Leal et al., 2013). By contrast, transcript levels for the ORs sensitive to DEET and 4EP, *CquiOR136* and *CquiOR99*, respectively, were much higher in stylets than in the labium (Figure 4). Next, we prepared samples by separating the fascicles into two groups, labrum vs. hypopharynx plus mandible plus maxilla. These qPCR analysis demonstrated that in general the tested receptors are enriched in labrum (Figure 5), except for *CquiOR1*, which showed high transcript levels in both types of tissue. Of particular note, transcripts for two receptors sensitive to compounds that elicit clear behavioral responses, *CquiOR136* and *CquiOR99*, respectively, were predominantly detected in labrum. Odorants that elicit clear behavioral responses (attraction, oviposition, repellency, etc) by activating a single receptor allow us to further interrogate the system with well-designed behavioral assays. For example, one could incapacitate the labrum and measure if/how the treatment affects behavior. For the design of such experiments it is important to know the location of the receptors within the labrum. Then we prepared samples by separating the labrum tip (cut at the same length as the labella) and the rest of the labrum, extracting RNA and an analyzing transcript levels by qPCR (Figure 6). Transcripts for all tested *OR* were abundant in both parts of the tissue given the existence of apical and subapical labral sense organs in female mosquitoes (Lee, 1974). More importantly, the data clearly indicated that *CquiOR99* and *CquiOR136*, among others, were more expressed in the tip of the labrum (Figure 6). We then designed experiments to test whether the expression of these two receptors in the labrum is essential for behavioral responses.

**Figure 7:**
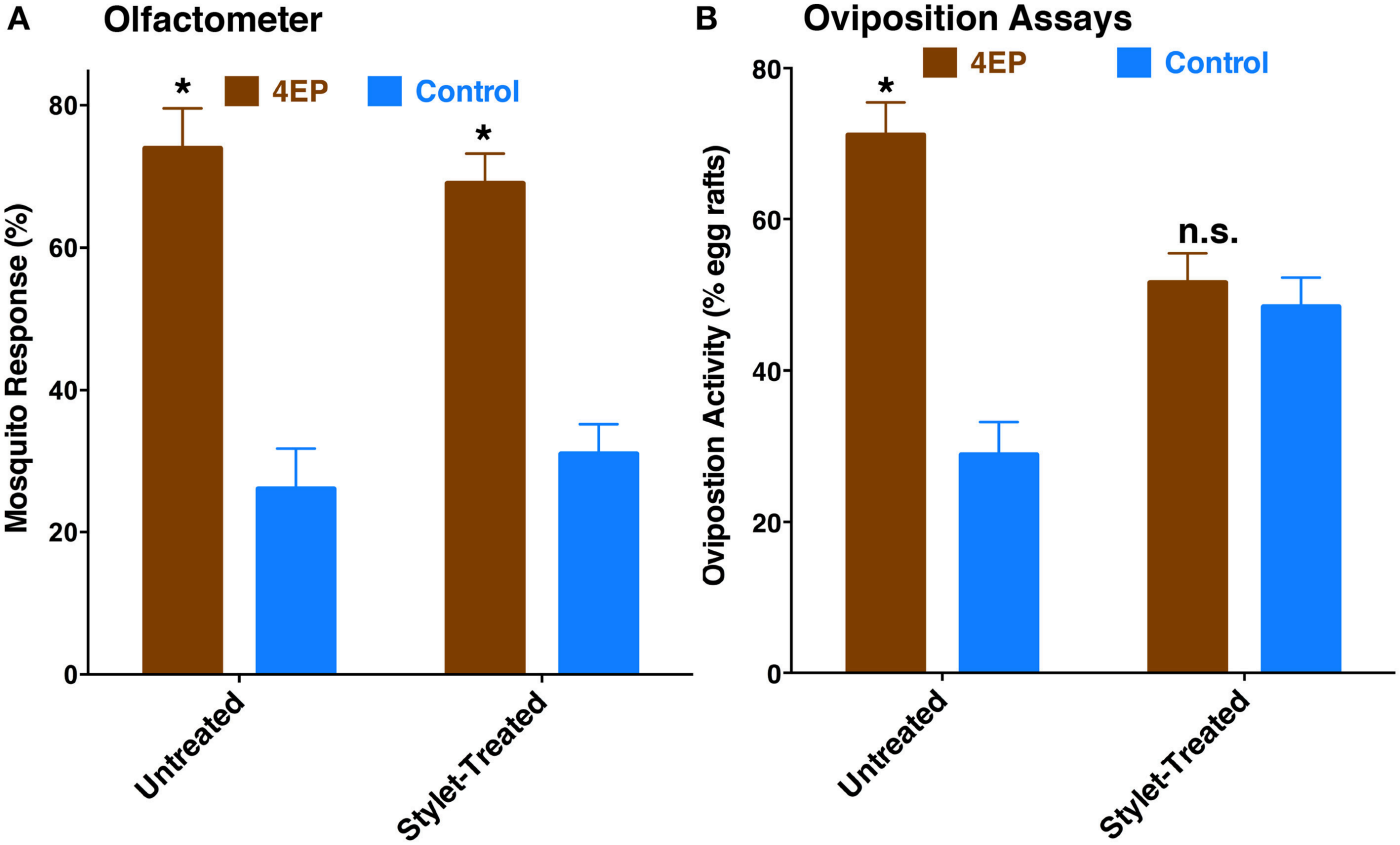
**Behavioral responses of gravid female mosquitoes to 4EP. (A)** In a dual-choice olfactometer, both females having their stylets coated with nail polish and untreated females were more significantly attracted to the arm of the olfactometer with 4EP (untreated, *P* = 0.016, *n* = 5; stylet-coated, *P* = 0.002, *n* = 8). **(B)** In oviposition bioassays, untreated female mosquitoes laid significantly more egg rafts in 4EP-treated cups (*P* = 0.002, *n* = 21), whereas the same treated females (stylet-coated with nail polish) used in olfactometer, showed no oviposition preference (*P* = 0.396) for 4EP. ^*^p ≤ 0.05.

**Figure 8:**
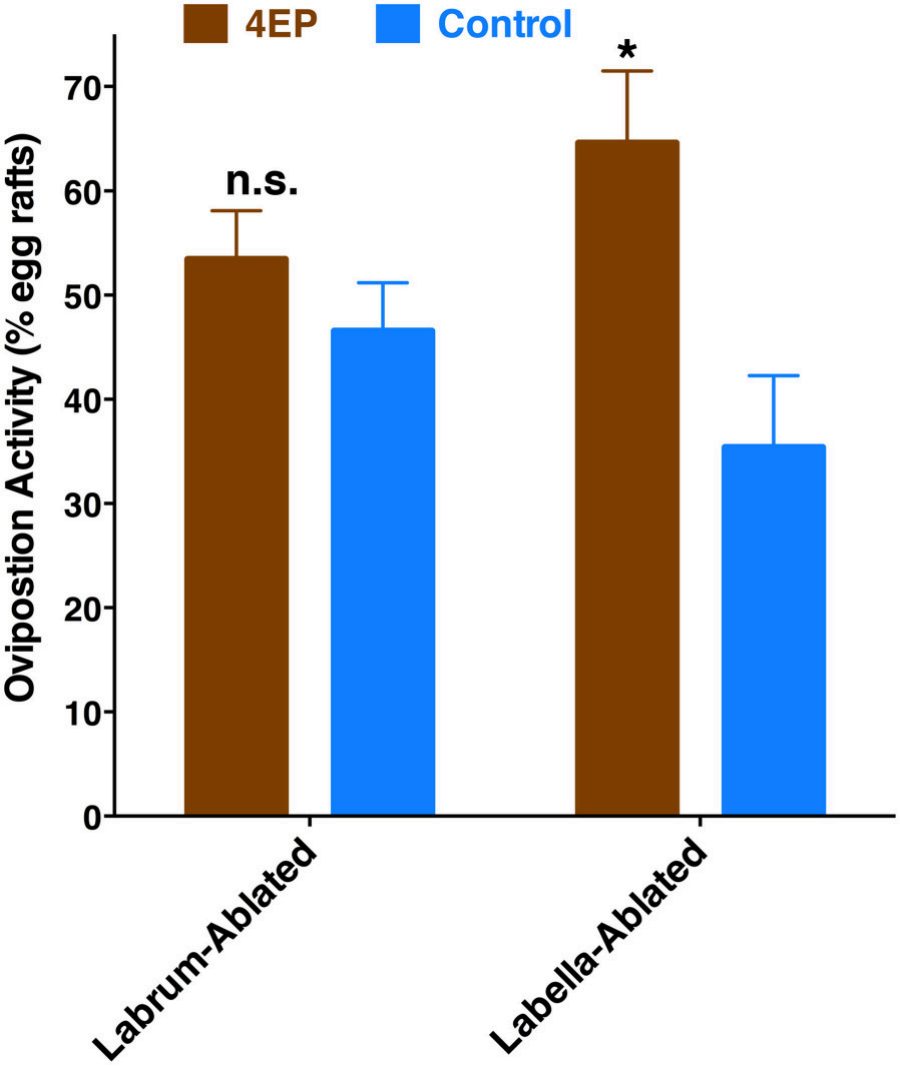
**Effect of microsurgery on 4EP-mediated oviposition**. While labella-ablated mosquitoes laid significantly more egg rafts in 4EP-treated cups (*P* = 0.002), labrum-ablated mosquitoes laid eggs indiscriminately in 4EP-treated and control cups (*P* = 0.25). (*n* = 27 in both experiments). ^*^p ≤ 0.05.

Previously, we have demonstrated that 4EP is both an attractant and an oviposition stimulant. First, gravid traps baited with 4EP collected significantly more female mosquitoes than control traps thus implying long-range and/or middle-range attraction. Secondly, in-door oviposition assays showed that significantly higher numbers of egg rafts are laid in water trays baited with 4EP than in control trays (Zhu et al., 2013). We then asked the question whether expression of CquiOR99 in the labrum is essential for attraction and/or oviposition behavior. Using a dual-choice olfactometer (Figure 2), we first tested whether gravid female mosquitoes were attracted to 4EP from a distance in the absence of water. Gravid female mosquitoes showed a clear preference for 4EP thus confirming that indeed 4EP acts at-a-distance (Figure 7A), as previously implied from captures of gravid females in field experiments (Zhu et al., 2013). Subsequently, we tested whether these mosquitoes would prefer to lay eggs in cups baited with 4EP as compared to control cups. As previously observed (Zhu et al., 2013), gravid females laid significantly more egg rafts in 4EP-treated cups than in control (Figure 7B). Next, we asked whether the stylet is involved in attraction at-a-distance and oviposition decisions. Previous knockdown experiments showed a direct link between CquiOR99 and oviposition behavior (Zhu et al., 2013), but we did not know then that CquiOR99 is also expressed in the proboscis. Given that tissue-specific knockdown of ORs is not yet feasible with mosquitoes, we applied a conventional approach, coating tissues with nail polish (Maeno et al., 2011; van Bergen et al., 2013), to interrogate whether sensory organs are involved in the reception of test odorants. Mosquitoes having the tip of the stylet coated with nail polish responded to 4EP in the dual-choice olfactometer in the same manner as the untreated mosquitoes (Figure 7A). We did not observe any significant effect of surgery on flight activity. The same mosquitoes in oviposition assays did not discriminate 4EP-treated from control cups (Figure 7B). Additionally, we prepared another group of mosquitoes by coating their labella (Figure 1) and conducted oviposition assays. Labella-coated mosquitoes laid significantly more egg rafts in 4EP-treated cups than in control cups (*n* = 28, 4EP, 9.5 ± 0.56; control, 6.75 ± 0.44; *P* < 0.001). Since the labella was insensitive to 4EP, we surmised that it could be used as a control in the microsurgery experiments. We then prepared two groups of mosquitoes: one group with the tip of the labrum excised and another group with the labella removed, and their response to 4EP were examined in oviposition assays. While the labella-ablated mosquitoes laid significantly more eggs in 4EP-treated than in control cups, this behavior was suppressed in labrum-ablated mosquitoes (Figure 8). Taken together, these data suggest that the tip of the labrum, but not the tip of the labium (labella; Figure 1), is involved in short-range reception of 4EP.

**Figure 9:**
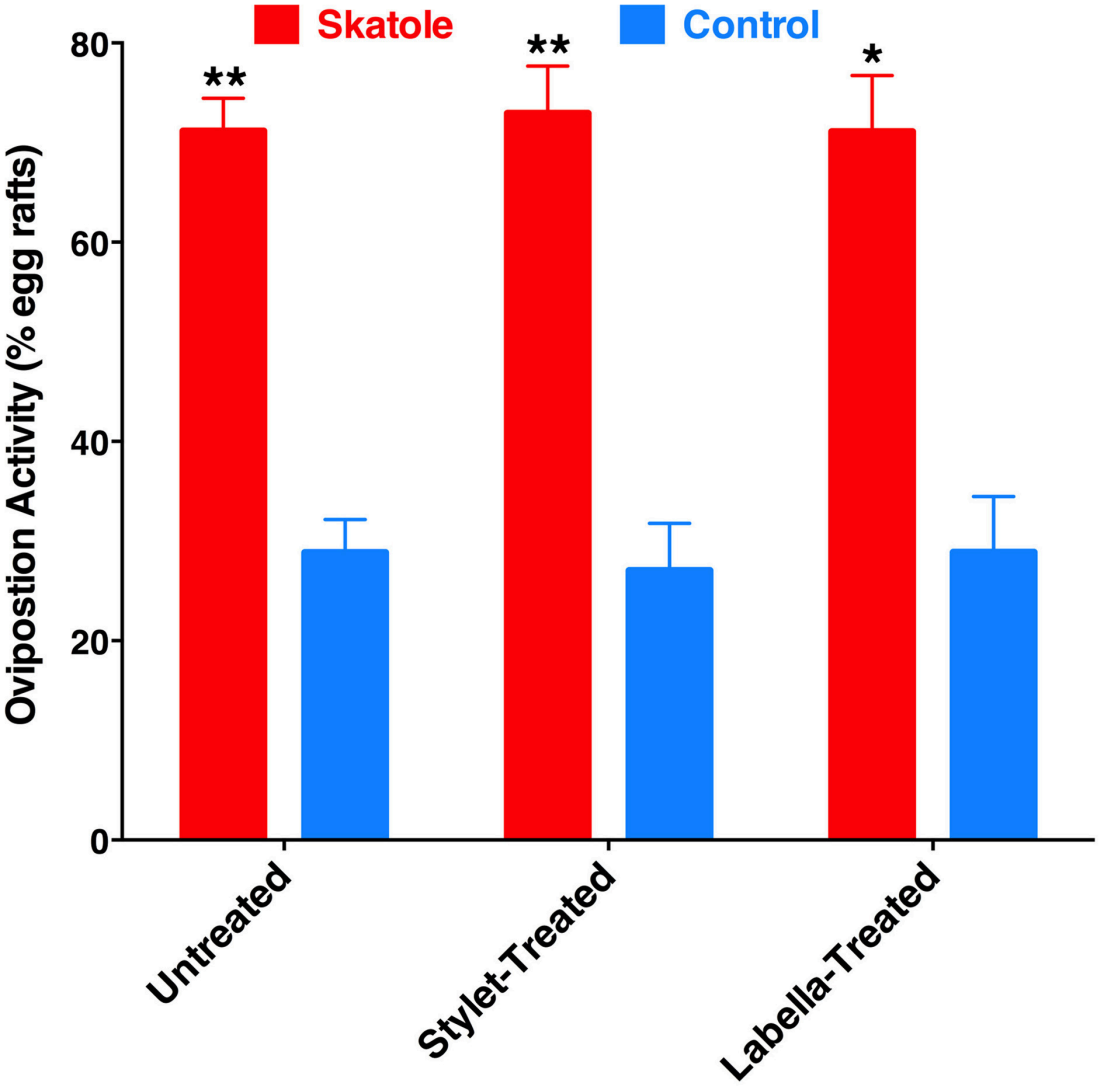
**Effect of coating on skatole-mediated oviposition**. Mosquitoes with the stylet coated with nail polish laid significantly more eggs rafts in skatole-treated cups (*P* = 0.0001; *n* = 11), as did untreated mosquitoes (*P* < 0.0001, *n* = 16). Mosquitoes with the labella coated with nail polish also showed preference for skatole-treated cups (*P* = 0.039, *n* = 8). ^*^p ≤ 0.05; ^**^p ≤ 0.01.

To test whether reception of oviposition attractants by the labrum is a common feature of mosquito biology, we performed oviposition assays using skatole, another oviposition attractant with demonstrated activity in field experiments (Mboera et al., 2000; Leal et al., 2008). Our rationale was that if this would be a common feature of mosquito biology, it would be manifested by all oviposition attractants. We compared in our indoor bioassays the effect of skatole on oviposition by untreated gravid female mosquitoes vs. treated mosquitoes by having either the tip of the stylet or the labella coated with nail polish. Not surprisingly, untreated mosquitoes laid significantly more egg rafts in water cups treated with skatole than control water cups (Figure 9). Similarly, mosquitoes having their stylets or labella coated with nail polish showed a clear preference for skatole-treated cups (Figure 9). We, therefore, concluded that the proboscis is not involved in the short-range reception of the oviposition skatole, in marked contrast with our observations with 4EP. These findings, albeit not surprising given the trace amounts of *CquiOR21* found in proboscis (Figure 3), suggest that reception by the proboscis of compounds-eliciting oviposition is not a common feature of mosquito biology.

**Figure 10:**
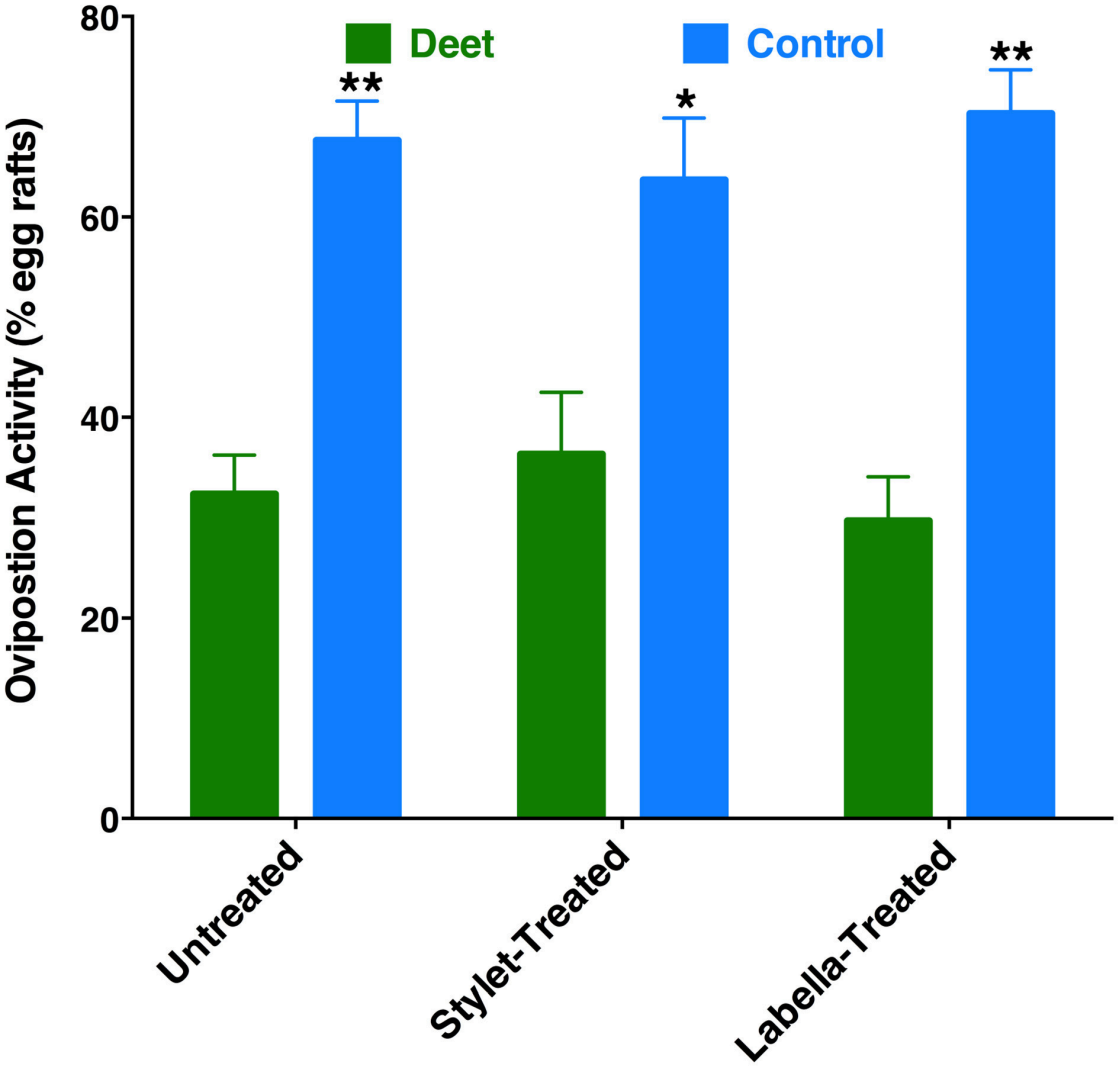
**Effect of coating on oviposition deterrence by DEET**. Untreated, stylet-coated, and labella-coated mosquitoes laid significantly more eggs in control cups than in cups treated with DEET (*P* = 0.0001, *n* = 10; *P* = 0.0038, *n* = 8; *P* = 0.0002, *n* = 6, respectively). ^*^p ≤ 0.05; ^**^p ≤ 0.01.

Recently, it has been demonstrated that DEET has oviposition deterrent activity (Tikar et al., 2014). Considering the significant expression of a DEET receptor, CquiOR136, in the labrum (Figure 5), we speculated whether reception at the proboscis might be important for this deterrent activity. First, we confirmed in our dual-choice bioassays that when gravid mosquitoes are given the choices of water cups, one treated with DEET vs. control (untreated cup), females laid significantly more egg rafts in control cups (Figure 10). Next, we performed these experiments using female mosquitoes whose stylets or labium have been coated with nail polish. Likewise, a deterrent effect of the DEET-treated cups was observed when the ability of reception by the proboscis was suppressed (Figure 10). These findings imply that reception of DEET by the antennae is necessary and sufficient for deterrence in oviposition cage assays.

**Figure 11:**
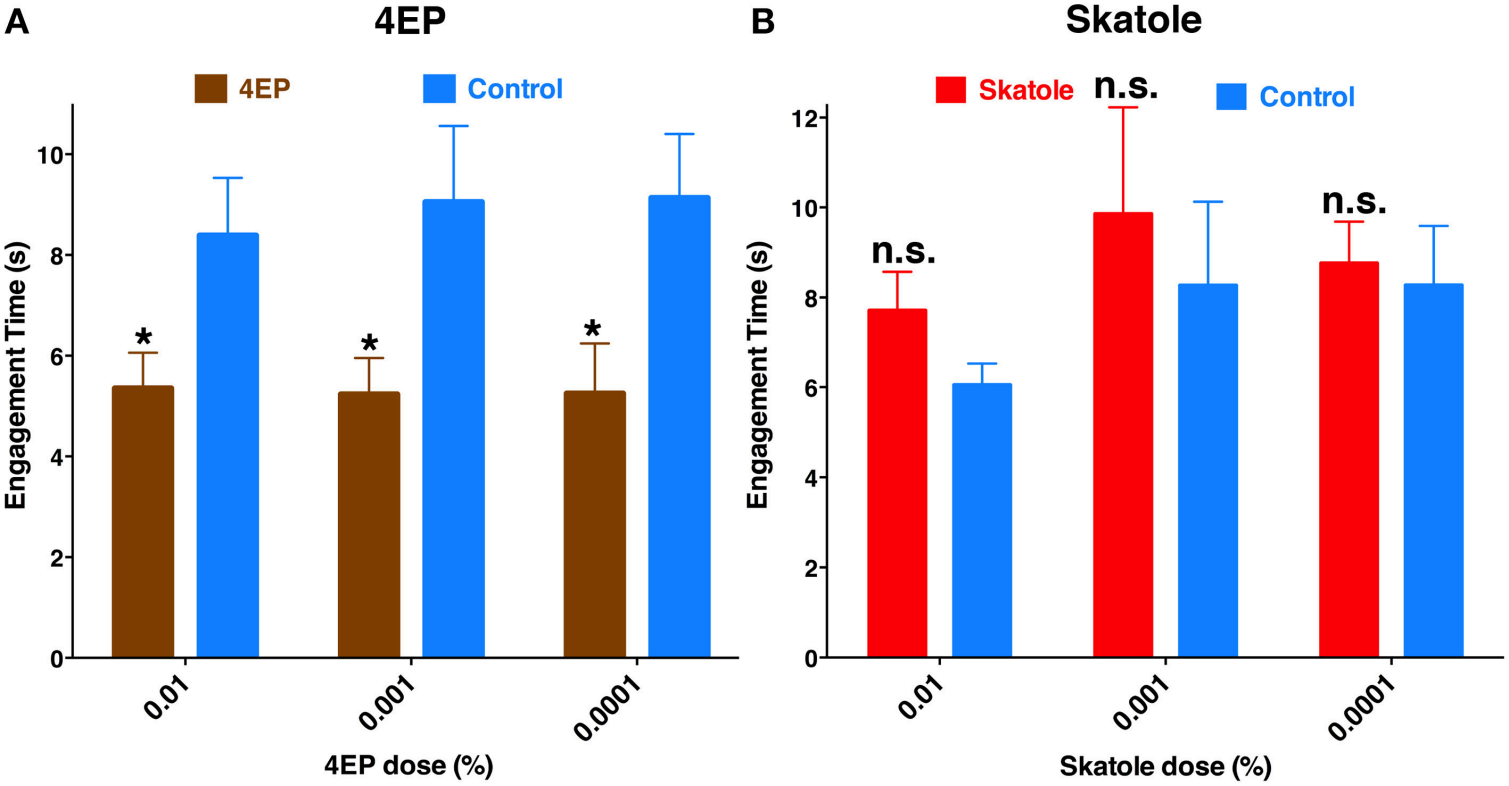
**Chemical-mediated shortening of engagement time**. In an arena designed to mimic a human arm, blood-seeking mosquitoes were given physical and chemical stimuli, and a choice to feed either on blood treated with an additional chemical signal (4EP or skatole) or untreated blood. **(A)** The duration of probing before continuous blood-feeding started, i.e., the “time of engagement” was significantly reduced with blood was treated with 0.01, 0.001, and 0.0001% of 4EP (*P* = 0.02; *n* = 31–40; *P* = 0.04; *n* = 17–19; *P* = 0.02, *n* = 15–16, respectively). **(B)** By contrast, skatole at the same concentrations did not affect the time of engagement (*P* = 0.14, *n* = 21–28; *P* = 0.63, *n* = 11–14; *P* = 0.15; *n* = 29–46, respectively). ^*^p ≤ 0.05.

If odorant reception by the labrum is important for probing and blood feeding, then the presence of these odorants should have a demonstrable impact on these behaviors. Considering that 4EP is a constituent of human blood (Loke et al., 2009) and transcripts of a 4EP receptor, *CquiOR99*, were abundant in the tip of the labrum (Figure 6), we designed a modified surface-landing and feeding bioassay to address this question. To mimic a human arm, a Dudley tube, internally painted with black craft enamel and was placed inside the arena. Water at human blood temperature was circulated through the tube. Thus, the Dudley tube provided physical stimuli (color and temperature). Two syringe needles, one on the top and the other on the bottom of the tube, supplied a common attractant, CO_2_. Two cotton rolls were inserted in-between the needle and the Dudley tubes. Both cotton rolls were loaded with defibrinated sheep blood, one of them being spiked with 4EP (treatment) and the other used as a control. We recorded the experiments and retrieved the tapes to focus mainly on probing behavior. Specifically, we compared the times of engagement (see MandM). It is worth mentioning that the engagement time is not the time to start probing, neither the duration of feeding. To determine the possible effect of the stimulus (4EP), we paid close attention to one individual at a time, and measured the time each female spent probing and when she started feeding. Our rationale is that a phagostimulant or another type of signal would reduce this time period, which we named “time of engagement.” Mosquitoes with coated or ablated stylets do not probe and feed, and therefore all mosquitoes used in this set of measurements were unaltered. The time of engagement was significantly shorter in blood containing 4EP at concentrations 0.01, 0.001, and 0.0001% than in their respective controls (Figure 11). By contrast, there were no significant differences in the times of engagement when blood was treated with skatole at the same concentrations. Of particular note, as opposed to 4EP, skatole is not a common blood constituent (Loke et al., 2009).

Taken together, these data suggest that reception of 4EP by CquiOR99 expressed in the labrum is important not only for oviposition decisions, but also for reducing probing and initiation of blood feeding. Thus, the labrum plays multitasking roles, including its mechanical nanosharp tips in skin penetration (Kong and Wu, 2010), a conduit for suction of blood, decision making in at least 4EP-mediated oviposition, and, most importantly, reduction of probing duration. At the time of this writing, Won Jung and collaborators (Jung et al., 2015) elegantly demonstrated that olfactory receptor neurons in the stylet of *Aedes aegypti* are involved in blood feeding behavior. Interestingly, our research group reached the same conclusion by using entirely different approaches. Our research provides additional evidence suggesting multitasking roles of the labrum in 4EP-mediated blood feeding as well as oviposition behaviors.

## Author contributions

WL conceived the research and designed the experiment; YC, GB, KT, and WL performed experiments; YC, GB, and WL analyzed the data; WL wrote the manuscript with input from YC and GB.

## Funding

Research reported in this publication was supported by the National Institute of Allergy and Infectious Diseases of the National Institutes of Health under Award Number R01AI095514. The content is solely the responsibility of the authors and does not necessarily represent the official views of the National Institutes of Health.

### Conflict of interest statement

The authors declare that the research was conducted in the absence of any commercial or financial relationships that could be construed as a potential conflict of interest.
